# Housing, health and energy: a characterisation of risks and priorities across Delhi’s diverse settlements

**DOI:** 10.1080/23748834.2020.1800161

**Published:** 2020-09-10

**Authors:** Emily Nix, Jonathon Taylor, Payel Das, Marcella Ucci, Zaid Chalabi, Clive Shrubsole, Michael Davies, Anna Mavrogianni, James Milner, Paul Wilkinson

**Affiliations:** a UCL Institute for Environmental Design and Engineering, University College London, London, UK; b Department of Civil Engineering, Tampere University, Tampere, Finland; c Department of Physics, University of Surrey, Guildford, UK; d Department of Public Health, Environments and Society, London School of Hygiene and Tropical Medicine, London, UK; e Air Quality & Public Health Group, Environmental Hazards and Emergencies Dept, Centre for Radiation, Chemical and Environmental Hazards, Public Health England, Chilton, UK; f Centre on Climate Change and Planetary Health, London School of Hygiene & Tropical Medicine, London, UK

**Keywords:** Housing, health risks, energy use, Delhi, India, intervention priorities

## Abstract

Improved housing has the potential to advance health and contribute to the Sustainable Development Goals. Research examining housing, health and energy use in low-income countries is limited; understanding these connections is vital to inform interventions for healthy sustainable human settlements. This paper investigates the low-income setting of Delhi, where rapid urbanisation, a varied climate, high pollution levels, and a wide variation in housing quality could result in significant energy use and health risks. Drawing on approaches from health and the built environment and existing data and literature, a characterisation of energy use and health risks for Delhi’s housing stock is completed. Four broad settlement types were used to classify Delhi housing and energy use calculations and health risk assessment were performed for each variant. Energy use is estimated to be nearly two times higher per household among planned housing compared with other settlement types. Health risks, however, are found to be largest within informal slum settlements, with important contributions from heat and particulate matter across all settlements. This paper highlights intervention priorities and outlines the need for extensive further research, particularly through data gathering, to establish evidence to accelerate achieving healthy, sustainable and equitable housing in Delhi.

## Introduction

Better housing has the potential to improve health and well-being (Howden-Chapman *et al*. 2012), advance development, especially in low-income countries (Haines *et al*. 2013), and support environmental objectives, notably in relation to energy consumption (Haines *et al*. 2009). Housing interventions have been shown to improve physical and mental health (Thomson *et al*. 2001, Office of the Deputy Prime Minister 2006, Gibson *et al*. 2011, Howden-Chapman *et al*. 2011) and reduce health inequalities (Thomson *et al*. 2013). Energy efficiency in housing is vital for climate change mitigation objectives, with buildings currently accountable for 38% of total global energy usage and 25% of energy-related CO_2_ emissions; hence, energy efficiency in homes is crucial to reduce emissions (Wilkinson *et al*. 2007). Improved housing, therefore, has an important role in achieving the United Nations Sustainable Development Goals, particularly Goal 11: Make cities and human settlements inclusive, safe, resilient and sustainable, but also to achieve energy efficiency (Goal 7), combat climate change (Goal 13) and ensure health and well-being for all (Goal 3) (United Nations Development Programme 2015).

Research examining the connections between housing, health risks and energy use in housing has tended to focus on high-income countries with temperate climates that have adopted energy efficiency targets. Research has evaluated the health impacts of strategies and policies for energy efficiency in housing. In the UK, for example, the implementation of insulation in homes has been shown to offer protection against cold-related mortality (Wilkinson *et al*. 2007). However, energy efficiency interventions may also cause unintended adverse health impacts (Shrubsole *et al*. 2014), without due consideration (Davies and Oreszczyn 2012). For example, both modelling (Milner *et al*. 2014) and empirical measurements (Symonds *et al*. 2019) of UK housing indicate energy efficiency improvements are likely to be responsible for increases in indoor radon levels. The health benefits of housing interventions in low-income countries is considered much greater (Wilkinson *et al*. 2009), yet there is a little evidence on the links between energy, health, and housing in these developmental and climate contexts – more research is necessary to understand risks and priorities for interventions in these different settings. Housing quality in low-income countries can be extremely varied, and interventions are vital to achieve energy efficiency targets and health goals simultaneously.

This paper aims to investigate housing health risks and energy use in the context of a low-income country and identify where interventions are needed. The city of Delhi, India, was selected as a case study, as it provides an example of a rapidly urbanised city, with unprecedented levels of uncontrolled housing development, which may present significant challenges in providing sustainable and healthy living environments.

### Background

India is projected to be the most populous country by 2050, predicted to be home to 20% of the world’s population with nearly 1.7 billion inhabitants, and with the urban proportion expected to grow from 31% to 52% during the next four decades (United Nations 2017). Pressures from this rapid growth, along with a disorganised approach to housing provision (Mahadeva 2006), can be seen through a shortage in housing and related infrastructure across Indian cities (Sivam and Karuppannan 2002). The population of the National Capital Territory (NCT) of Delhi has substantially increased over the last century from just under 1 million inhabitants in 1941 to over 16 million in 2011 (Government of India 2011). This growth has coincided with the development of unauthorised and informal settlements, where the slum population is reported to account for 47% of the housing stock (Government of National Capital Territory of Delhi 2008). These settlements suffer from poor quality housing, cramped spaces and a lack of basic services and infrastructure (Goli *et al*. 2011), with significant risks of infection and injury (Ezeh *et al*. 2017).

With this rapid growth, there are also energy challenges. In 2015, India was the fourth-largest energy consumer (after the United States, China and Russia), with energy consumption rates growing annually (U.S. Energy Information Adminstration 2018). International Energy Agency (IEA) Energy Balance Statistics for India state that the residential sector accounts for the largest proportion (38%) of the country’s energy use (International Energy Agency 2009). Although per capita energy consumption remains very low, future projections indicate increased electricity use and higher ownership of appliances as incomes increase (Reddy and Srinivas 2009), resulting in higher CO_2_ emissions (Rout 2011, van Ruijven *et al*. 2011). Within Delhi, the residential sector is responsible for 45% of electricity sales (Government of National Capital Territory of Delhi 2013). Consequently, this combination of increased appliance usage and housing growth could result in high future demands on energy consumption.

Environmental conditions in Delhi are extremely challenging. Delhi experiences a composite climate (Bureau of Indian Standards 2005), with a large seasonal variation between a cold winter (mean minimum temperatures below 10°C), dry and hot summer (highs up to 45°C) and a humid monsoon period (Indian Society of Heating Refrigerating and Air-Conditioning Engineers). Temperatures are predicted to increase by 3–4°C by 2100 due to a changing climate (Defra, Akhtar 2007, Singh and Dhiman 2012), with heat waves becoming more frequent, risking significant impact on energy consumption through air conditioning (A/C) use (Sivak 2009, Akpinar-Ferrand and Singh 2010) and heat-related mortality (Akhtar 2007). A humid monsoon season coincides with an outbreak of mosquitoes, with vector-borne disease epidemics becoming more likely (Dhiman *et al*. 2010). Outdoor air pollution levels are notoriously high due to generation from vehicles, industry, diesel generators, and brick kilns (Guttikunda and Goel 2013). Delhi’s mean annual concentrations of particulate matter PM_2.5_ (PM with an aerodynamic diameter ≤2.5 μm) regularly exceed 100 μg/m^3^ (Government of National Capital Territory of Delhi 2013), severely breaching World Health Organization (WHO) air quality guidelines of 10 μg/m^3^ (WHO 2006, 2010). Due to both anthropogenic (waste burning for heating) and meteorological conditions, PM_2.5_ winter levels are two to three times higher than summer and monsoon periods (Guttikunda and Gurjar 2012). Furthermore, the effects of rapid urbanisation have resulted in polluted water supplies (Ministry of Environment and Forests Power Government of India 2001), poor solid waste management (Talyan *et al*. 2008) as well as heightened noise pollution (Firdaus and Ahmad 2010). These external factors will have a substantial influence on indoor conditions, and hence household energy consumption and potential health impacts.

### Objectives

Rapid urbanisation, significant informal housing provision, increased energy use in the domestic sector, along with a challenging external environment, suggests substantial sustainability and health risks across Delhi’s housing. There are, however, opportunities for interventions that could help meet energy and health goals simultaneously. This paper aims to make a broad assessment of health risks and energy use across Delhi’s housing to inform priorities for interventions that could improve health and sustainability. As such, this paper aims to answer the following questions:

- What are the housing characteristics of Delhi’s housing stock?
- What are the energy use characteristics and principle health risks, and how do these differ across the housing stock?
- What are the priorities for housing interventions to advance health and sustainability goals?

An assessment of Delhi’s housing, of this scale and type, has never been completed. Such work is necessary for identifying the key risks and priorities across Delhi, this will help inform avenues for further research as well as pathways for interventions, which then can be utilised by planners, engineers and architects to enable a transition towards a healthy sustainable urban environment.

## Methodology

**Figure 1:**
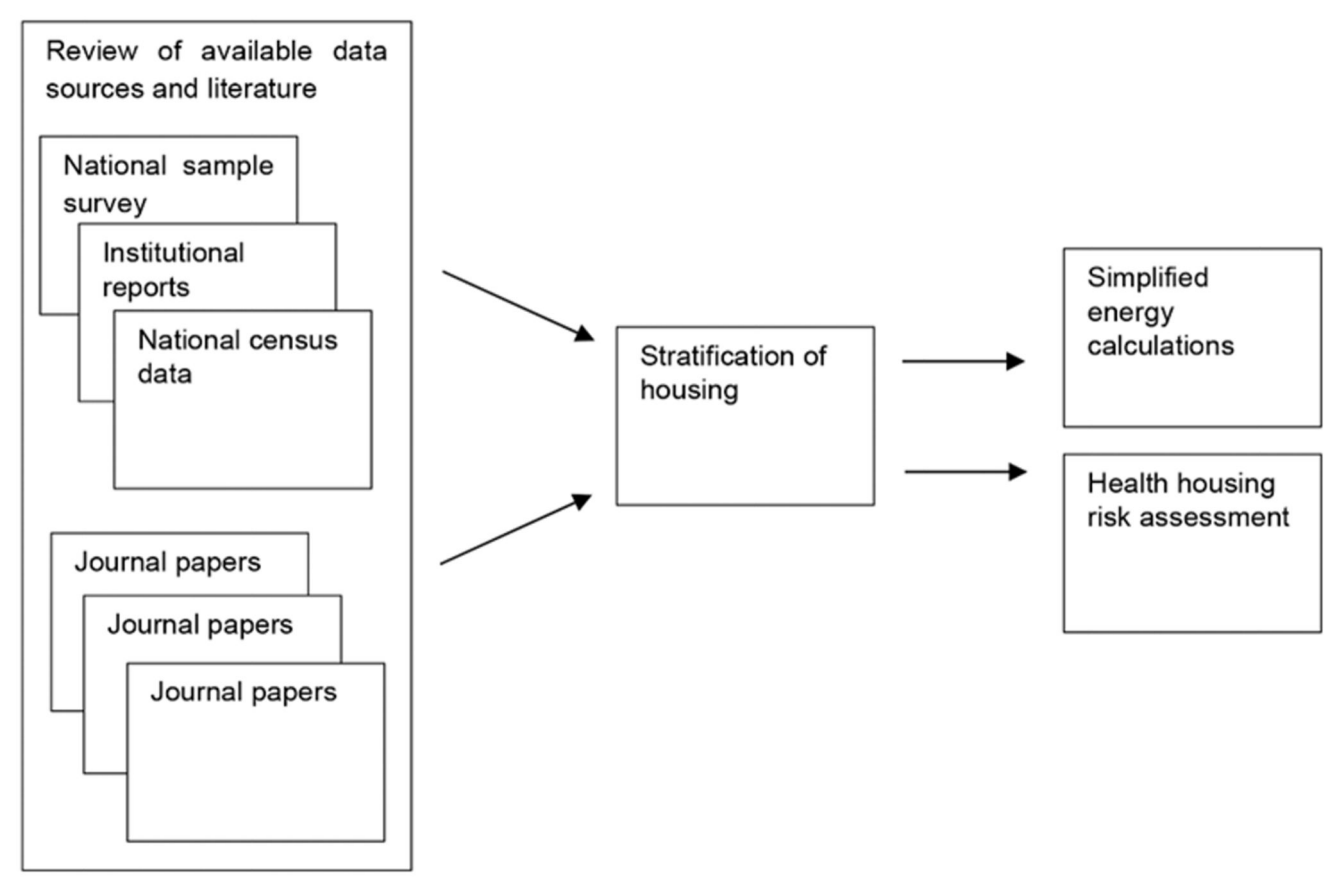
Overview of the methodology.

The methodology developed was informed by the fields of public health and the built environment. The work draws on existing data sets and available evidence and applies broad assessments to understand current energy use and health risks across Delhi’s housing. An overview of the approach used is shown in Figure 1.

### Stratification of Delhi’s housing stock

Housing stock models have been widely used in studies assessing city residential energy consumption and potential interventions, with the housing stock generally broken into distinct archetypes based on relevant housing surveys (Kavgic *et al*. 2010). Stratification of the housing stock is useful for both the development and assessment of policies and strategies that can improve health or reduce energy consumption in the given area. We aimed to develop a stratification method of Delhi housing to estimate current energy use and principle health hazards to develop and assess potential strategies.

For the case of Delhi, there is no comprehensive survey that details housing characteristics at the level needed to generate distinct archetypes. The India Housing Census (Government of India 2011) and Housing Condition National Sample Surveys (NSS) (N. S. S. O.-M. of S. & P. I. Government of India 2010a) provide basic details of common construction materials, floor areas, and the number of rooms per dwelling but do not provide detailed data on the built form, such as thermal properties, layouts, or ventilation provision necessary to generate a set of arche-types. The annual Delhi Economic Survey provides a breakdown of dwellings by settlement type in Delhi (Government of National Capital Territory of Delhi 2008) (Table 1). These settlements follow three modes of development: informal, organic and formal, which have different planning jurisdictions. Formal housing areas are planned by governing development authorities or private agencies; these have formal legal sanction prior to development and should comply with building regulations (Sivam 2003). Informal housing is composed of unauthorised colonies, built illegally on private land, and slum settlements, both of which lack legal tenure (Sivam 2003). Organic settlements consist of old urban housing and traditional rural villages, which have evolved over time (Ishtiyaq and Kumar 2011).

The modes of development have a significant influence on the built form and infrastructure characteristics and are connected with different income groups. These distinct modes of development were used as a basis to stratify Delhi housing stock into four settlement types as followed:

(1) High-income planned housing – these are developed by private agencies or the Delhi Development Authority (DDA), and often takes the form of plotted housing and multistorey flats that comply with building standards and have infrastructure provision (Sivam 2003, UN Habitat).
(2) Villages (including both rural and urban villages) – these have become part of Delhi with urbanisation (Ishtiyaq and Kumar 2011). These are more traditional in style – commonly open-fronted housing with 3–4 storeys, closely packed on narrow streets, and with little natural lighting (Kumar Soni 2011). They lack planned services, are suffering from overcrowding and dilapidation (Ishtiyaq and Kumar 2011), and tend to be occupied by mid- to low-income groups (UN Habitat).
(3) Unauthorised colonies (of which 13% are now regularised and given formal land rights), which are built illegally on agricultural land; information about these housing types is sparse. Infrastructure is provided through regularisation.
(4) Slum or locally known as *jhuggi jhopdi* (JJ) cluster settlements. These are home to the urban poor and are self-built, simple structures without land tenure, which undergo incremental growth with time (Sivam 2003). They are small and tightly cramped dwellings with floor areas no larger than 20 m^2^ (Ahmad and Choi 2011), suffering from a lack of services, inade-quate ventilation, and poor thermal comfort (Mitchell 2010).

**Figure 2:**
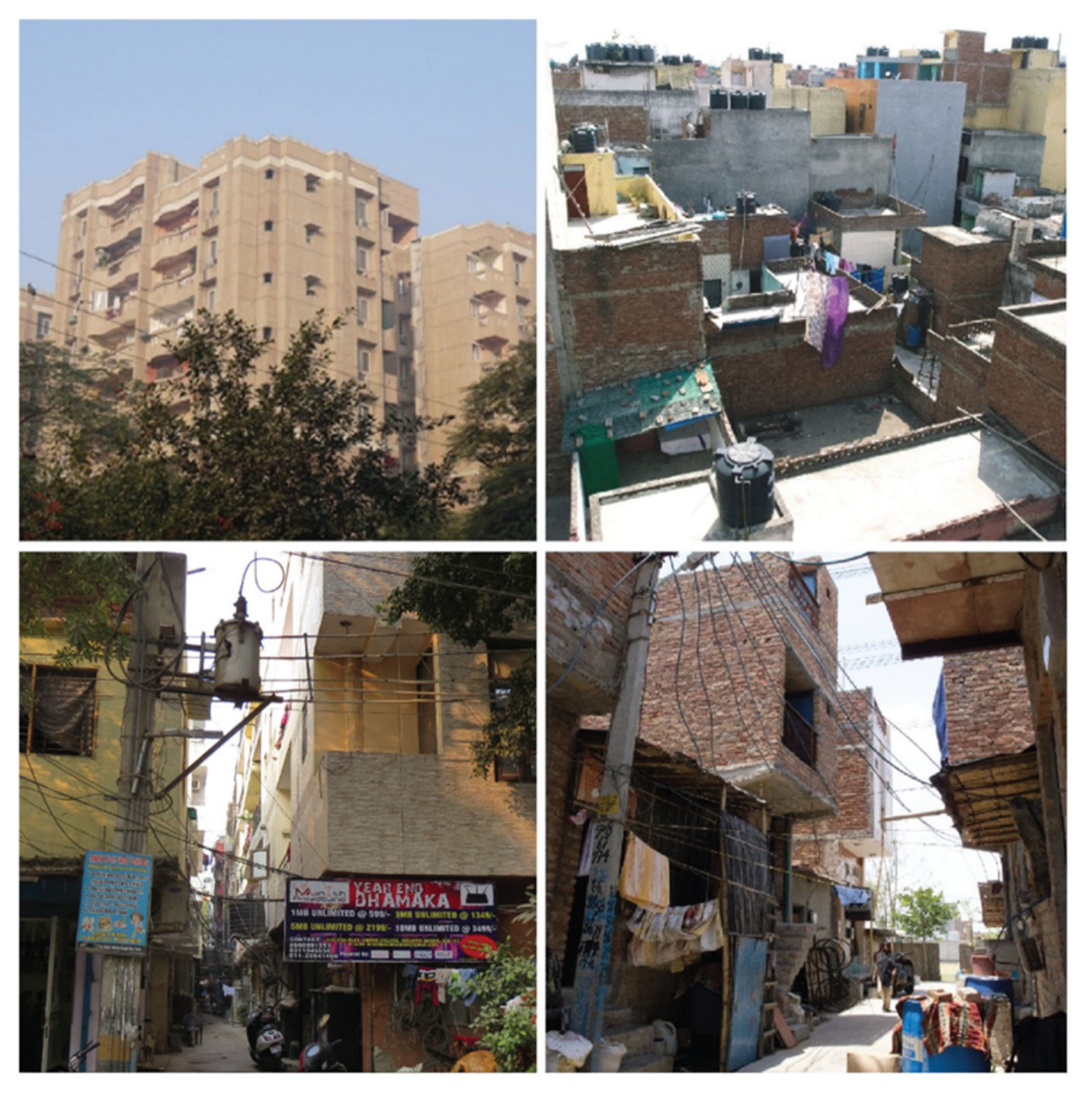
Examples of category 1: planned housing (top left), category 2: urban/rural villages (top right), category 3: unauthorised housing (bottom left), and category 4: JJ Clusters (bottom right).

Example photographs of the four categories of settlement types are shown in Figure 2. The various data sets were then linked to the settlements types, by household income, to develop a broad description of the housing in each category. This was cross-checked through field visits and personal correspondence with experts in housing in Delhi.

### Construction and energy use characteristics of settlement types

Dwelling construction materials and energy uses were linked to settlement types via several different sources. Here, we review the available data sources across the settlement types.

#### Construction characteristics

The Census and NSS show that in the case of all settlement types, the majority of housing is constructed with burnt brick (N. S. S. O.-M. of S. & P. I. Government of India 2010a, Government of India 2011). In the higher income group concrete accounts for 97% of all roofing material (N. S. S. O.-M. of S. & P. I. Government of India 2010a). Details of the material composition used in planned dwellings were based on studies from Kumar & Suman (2013), Ramesh *et al*. (2010), as well as the IT Toolkit EnEff ResBuild India (TERI and Fraunhofer Institute for Building Physics). Little information is available about composition in the unauthorised and urban village settlements and therefore we assume they are similar to the planned dwellings. The material used in the JJ settlements is likely to be much more varied. For example, concrete accounts for less than 50% of roof material in the lowest income group, with metal sheets, stone, canvas, or timber as other predominant materials (N. S. S. O.-M. of S. & P. I. Government of India 2010a).

#### Energy use and appliances

Both the Census and Housing Condition NSS surveys detail access to electricity and the use of primary cooking fuels. All settlements are likely to use Liquid Petroleum Gas (LPG) as the predominant cooking fuel (Government of India 2011). The majority of households have access to ceiling fans (TERI 2007) (N. S. S. O.-M. of S. & P. I. Government of India 2010b) and the penetration of appliances such as TVs and fridges are similar across settlement types. 99% of houses in Delhi have electricity for lighting (N. S. S. O.-M. of S. & P. I. Government of India 2010a). However, the use of A/C or air coolers is skewed towards high-income settlements, most likely planned dwellings. Ownership of A/C or coolers increases with the monthly per capita expenditure (MPCE) class from 26% of house-holds in the lowest decile to 77% in the highest (N. S. S. O.-M. of S. & P. I. Government of India 2010b). TERI reports higher ownership of A/C in higher housing tax bands with air coolers used predominately in mid-housing tax bands (TERI 2007). 48% of electricity sales in Delhi are domestic consumers (Government of National Capital Territory of Delhi 2013), and it is estimated that 50% of residential electricity use in summer months is due to a combination of ceiling fans, air coolers, and A/C (TERI 2007). The ownership of heating equipment is recorded to be low across all settlement types (TERI 2007).

The available data sources were mapped to the settlement types, these findings were confirmed by experts in the field with experience in the housing in Delhi. A summary of the characteristics of each settlement type can be found in Table 2.

### Assessment of household energy use

To assess typical energy consumption across the Delhi housing stock, we performed a simplified energy calculation to estimate energy use for each settlement types, supported by the data and housing characteristics gathered in the previous section. This method considers available data on the ownership of appliances (lighting, cooking, cooling and other end-use), average appliance power ratings, and time in use, and used assumptions on occupant behaviour based on available survey data. This simplified approach allows a broad estimation of energy use and CO_2_ per settlement type based on the currently available data. More detailed approaches, such as dynamic building energy simulations or the degree-day method, would require detailed data on building characteristics or a broader range of assumptions, which would require detailed surveying and measurements to perform at the stock level and was thus deemed beyond the scope of this exploratory work.

Total annual energy usage, *E*_*T*_, is taken to be the sum of the energy use of all appliances, based on the number of appliances of type *i, n*_*i*_, the power rating of appliance *i, P*_*i*_ and the time of use of appliance *i, t*_*i*_,:

(1) $$E_{T}=\underset{i}{\sum \;}n_{i}\times P_{i}\times t_{i}$$

CO_2_ emissions are then calculated by applying the appropriate carbon intensity coefficients for the fuels used and electricity generation. Energy use from cooking was taken to be the same across all settlement types, based on average LPG usage per month (D’Sa and Murthy 2004). Energy use for lighting and appliances was based on survey data assessing typical appliance use in residential dwellings in Delhi (TERI 2007). Hours of use of cooling appliances (fans, coolers and A/C in occupied bedroom and living rooms) was calculated to be the number of hours when the external temperature exceeded a threshold temperature. The threshold temperature was given by a thermal comfort study in composite climate in India (Indraganti 2011) and the external temperatures were taken from a typical weather file for Delhi (Indian Society of Heating Refrigerating and Air-Conditioning Engineers). Given the low ownership of heating appliances across all income groups, we did not consider this end-use type. Detailed inputs taken for appliance usage, power rating, and carbon intensity can be seen in Table 3. We carried out a sensitivity analysis for the planned settlement type to understand the impact of input variables on the output variable, described in Appendix A.

### Assessment of housing health risks

To characterise the distribution of health risks across the settlement types, a risk assessment was completed. Risk assessment techniques are widely established (British Standards Institution 2010) and have previously been used to assess housing health hazards elsewhere (Jacobs 2011). These use expert judgement to assess hazards, and generally consist of three steps; hazard identification, risk analysis and risk evaluation. Although more sophisticated methods exist, such as exposure-response relationships to calculate the disease burden, expert judgement has been a common method due to the lack of data, a wide range of potential hazards and multiple health outcomes. Studies using exposure-response relationships tend to have a narrow or single focus, such as those that review health risks from indoor temperatures (Scovronick and Armstrong 2012). We draw on existing frameworks but adapt them to the level of available data for Delhi.

### Hazard identification

Hazards identified for inclusion in the assessment are based on those included in the United Kingdom Housing Health Safety Rating System (HHSRS) (Sverdlik 2011), which is the most extensively developed assessment tool (Keall *et al*. 2010). As the context of Delhi significantly differs, we supplemented the UK HHSRS with additional hazards for particulate matter and vector-borne diseases which may be present in Delhi. The hazards assessed are listed in Table 4.

### Risk analysis methodology

A semi-quantitative method was used to characterise the principal health hazards. The method considers the like-lihood of occurrence and expected harm from available literature and data sources, experience from field visits and consultation with local experts. A consequence/probability matrix was developed to rank the risks (Table 5). Such methods are commonly used as a screening tool when many hazards are identified or where data is limited and can provide guidance on which hazards require further detailed analysis or should be treated first (British Standards Institution 2010). The likelihood of occurrence and the expected harm for each hazard was assessed to be either low, moderate, high or severe (or 1 to 4). The simplified hazard consequence/probability matrix was then used to rank the risks, giving a final score that was calculated by multiplying the likelihood of occurrence and expected harm. For further clarification, a definition of terms, the rationale for judgement, and assessment categories used are included in Appendix B.

The likelihood of occurrence and expected harm for each hazard for each settlement type in Delhi was based on a review of the academic literature (Appendix C). In particular, this included literature and datasets on:

- *Environmental exposure risk*; which includes evidence of outdoor environmental quality; indoor environmental quality; the level of infrastructure and services; and other related datasets;
- *Housing conditions/modifiers*; which considered risks in relation to the identified settlement types drawing on evidence of housing quality;
- *Health evidence*; which included relevant health data (recorded deaths in NCT of Delhi), EM-DAT data for India on mortality due to disasters (extreme heat and cold, fires, explosions, and collapse) and other relevant health studies.

Based on this evidence, all authors separately judged the likelihood of occurrence and expected harm as low, moderate, high or severe. These judgments were then compiled by taking the most common (mode) judgement (of the all the individual assessments) for the likelihood and expected harm, these were then used to calculate the final hazard rating. As the analysis method is largely subjective, combining the individual responses accounts for the variation between the authors’ ratings, this helps to improve the objectivity and rigour of the assessment. Mode, median, maximum and minimum hazard ratings results are provided in Appendix C for each hazard as a measure of the variability in ‘expert opinion’.

## Results

### Variation in household energy use and CO_2_ emissions

The highest energy use was estimated for the planned dwellings, mainly due to the high penetration of A/C (Table 6). Planned settlements dwellings were estimated to use between one half to a third more energy than dwellings from other settlement types. The lowest energy use is estimated in JJ clusters, where ownership of cooling appliances is low and space is limited. In planned housing cooling appliances were estimated to account for 44% of energy use, whereas in JJ cluster dwellings this is was found to account for less than 5% of energy use, with the majority of energy is used for cooking. This suggests that all energy needs are likely not be met in the JJ clusters, particular in regards to cooling.

**Figure 3:**
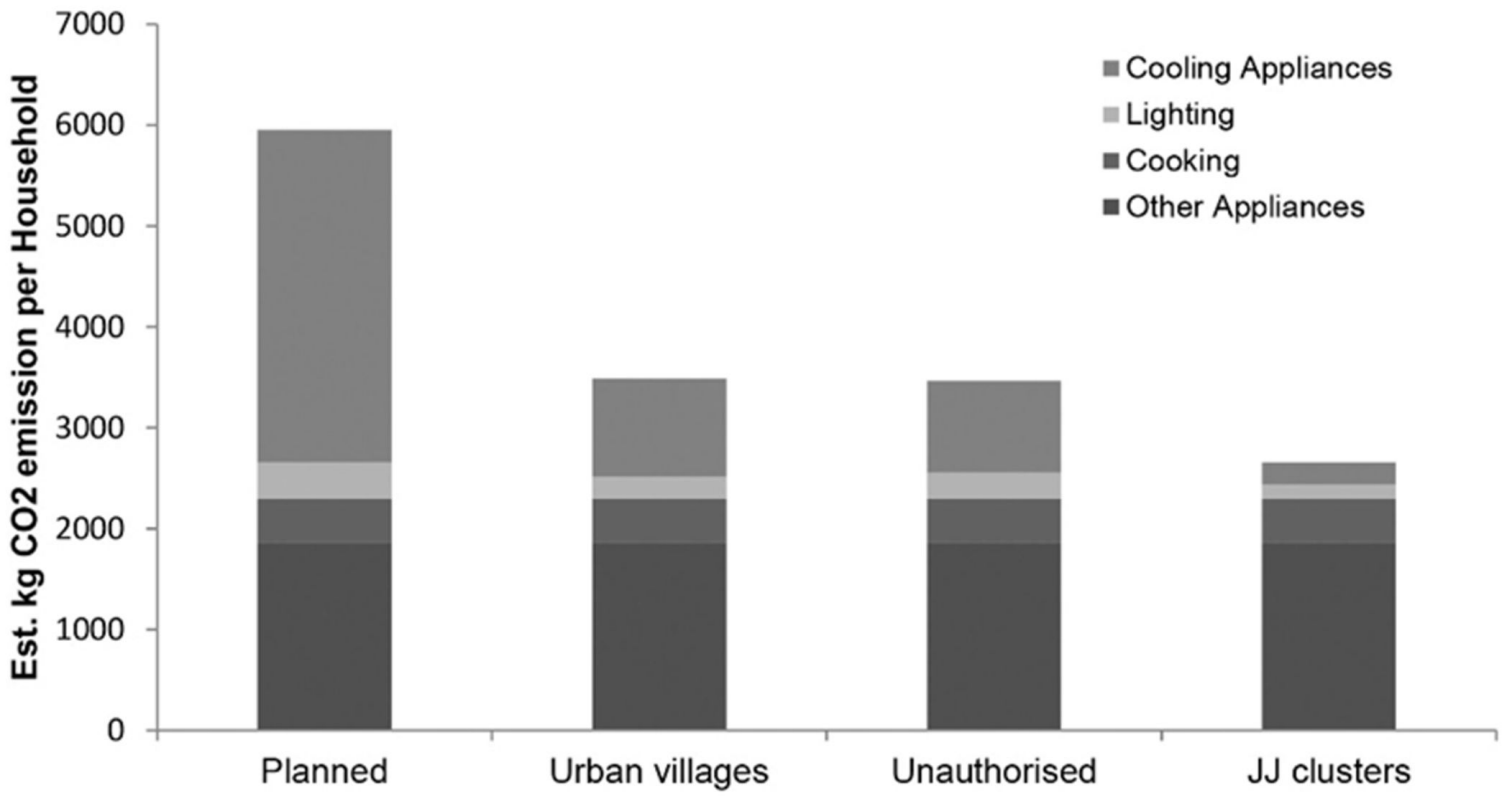
Estimated kg CO_2_ emissions per household by settlement type.

Estimated annual CO_2_ emissions (kg) per settlement type (Figure 3) are distributed similarly to energy consumption.

**Figure 4:**
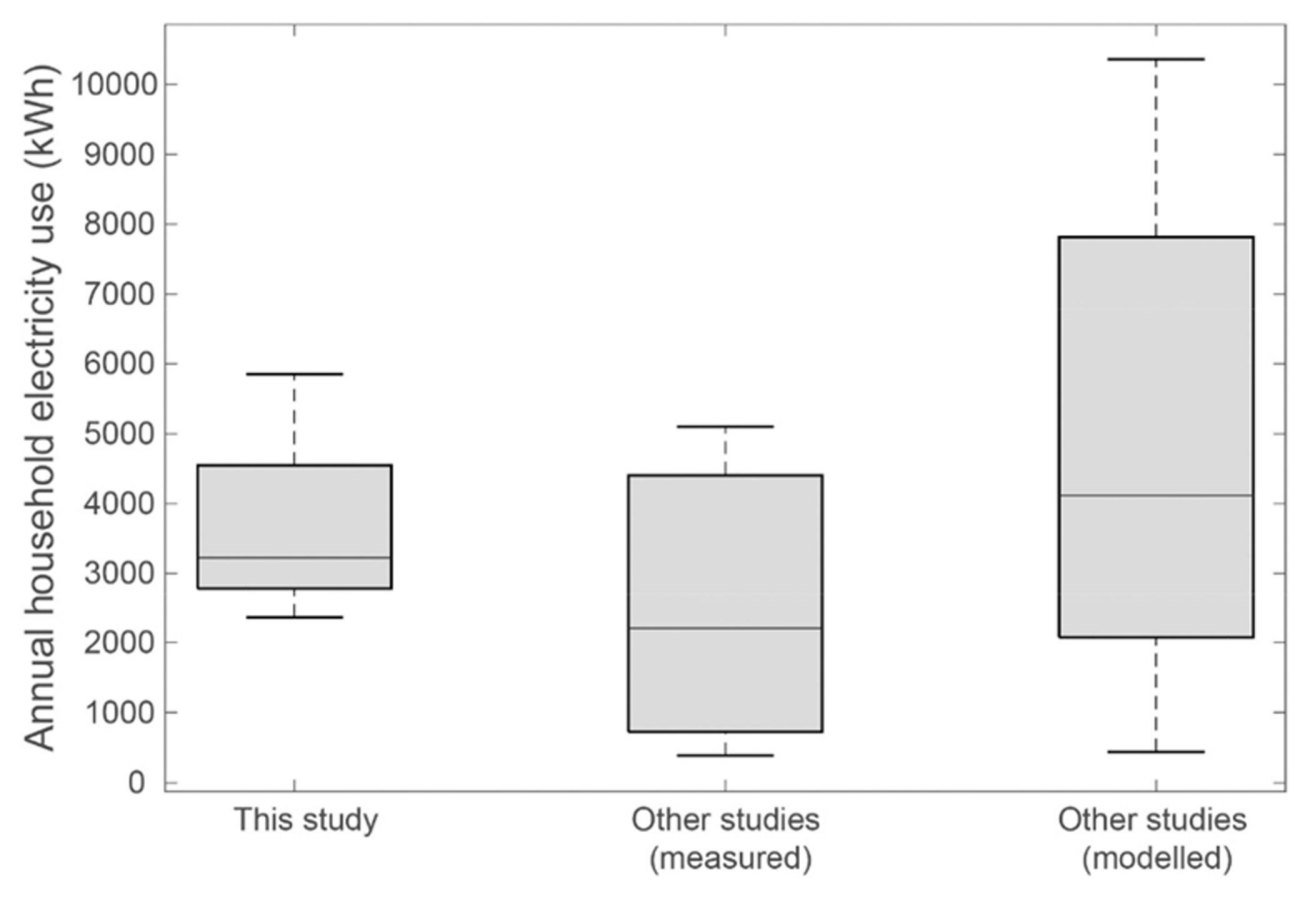
Comparison of electrical energy use estimates with other studies in literature in composite climate.

The Economic Survey of Delhi estimates residential electricity sales for 2011–12 to total 10,861GWh (Government of National Capital Territory of Delhi 2013). By scaling up to stock level, based on the distribution of settlement types and methods outlined above, we estimate a total annual consumption of 11,512GWh, an overestimate of 5%. This discrepancy could be due to a combination of simplifications and assumptions for each settlement type, in particular, the likely penetration of cooling appliances; the likelihood that total electricity use is not fully recorded due to illegal connections in JJ clusters; the use of back-up generators during blackouts; and the fact that A/C units might not be used for all hours that external temperatures exceed the specified threshold. The results of the estimated electricity use (i.e. without cooking) can be compared to other studies evaluating energy use in housing in a composite climate of India (Chunekar *et al*., Ramesh *et al*. 2012a, 2012b, 2013, Global Buildings Performance Network (GBPN) 2014, Praseeda *et al*. 2016, Mastrucci and Rao 2017). We find that the spread of results is broadly in line with our estimates (Figure 4). A sensitivity analysis of input parameters for the planned dwellings highlights power rating of A/C (R^2^ = 0.49) and hours of use (R^2^ = 0.33) are the most significant parameters for annual electricity use.

### Variation in housing health risks

The estimated hazard rankings for the different settlement types in Delhi can be seen below (Table 7). The scientific literature and datasets which were used by the authors to estimate the hazard risks and likelihoods are detailed in Appendix B. The final rankings were generated by taking the mode response for both likelihood of occurrence and the expected harm from the individual assessments. The completed risk analysis can now be used to prioritise which hazards require action first.

Particulate matter, heat and cold hazards were assessed to be the largest risks across all four categories of settlement, while vector-borne disease and water supply were also estimated to present significant risks to those in low-income JJ cluster settlements. Structural collapse, fire, overcrowding and, damp and mould hazards were estimated to be moderate risks across all settlements. JJ clusters were estimated to be the most ‘at risk’ settlement type, followed by urban villages and then unauthorised colonies. Planned settlements are likely to have high-quality dwellings and better access to services and infrastructure, hence providing the lowest risk environments. This variation in health risks across the settlement types presents is likely to cause a disproportional health burden on the low-income groups in Delhi.

## Discussion

This paper set out to provide an assessment of energy use and health risks across Delhi’s housing stock. The stock was divided into different settlement types, with data from a range of sources reviewed to estimate energy use characteristics and health risks across the settlement types.

### Priorities across settlement types

Our assessments indicate significant variation in energy use and health risks between the distinct settlement types found in Delhi. Planned dwellings were estimated to have a much greater consumption of energy in comparison to other settlement types, driven primarily by A/C usage. Taking an average occupancy of 5 across all settlement types gives 1170, 648, 642 and 471kWh/per person for planned, urban village, unauthorised and JJ cluster dwellings respectively, which illustrates that occupants in planned dwellings use almost use twice as much energy person compared to occupants from other settlement types. In urban/rural villages and unauthorised colonies, energy consumption was relatively low and poor indoor environmental conditions were the largest concern. Interventions should aim to improve indoor conditions but not significantly increase energy use. In the informal JJ clusters, strategies should focus on reducing a multitude of health hazards and improving dwelling quality as well as access to infrastructure, services and appliances. Given that the majority of energy use was from cooling appliances and household appliance (TVs and fridges), interventions should focus on energy efficiency for these appliances as well as passive cooling alternatives.

Largest risks to health were found to be hygro-thermal conditions (temperature and humidity) and air quality in all settlement types, hazards which could be reduced through better housing design and interventions to modify dwelling performance. Although housing quality has a significant impact on health risks, these are compounded further by levels of household income. For example; in the formally planned dwellings, the high penetration of A/C is likely to reduce exposure to high temperatures but then results in costly energy use, whereas in JJ clusters the poor dwellings and limited access to cooling appliances heighten health risks. This results in a huge disparity in energy use between settlement types, and thus the socio-economic development potential of populations living in those settlements. The strategies in each settlement will differ significantly and interventions will need to appropriately reflect the socio-economic status of each settlement type.

Opportunities to intervene in the planned dwellings are likely to be less restricted compared with the other settlement types, where interventions are limited by the crowded surroundings, dwelling size and financial capacity of the households. Policies will need to reflect current development mechanisms. In urban villages, strategies should focus on maintaining the quality of dwellings, as current regulations that do not restrict development have led to space partitioning and the reduction in ventilation and natural lighting. Interventions in unauthorised colonies can be incorporated in directives as unauthorised colonies become further regularised. In JJ clusters, interventions must be low cost (or heavily subsidised), easy to implement and employ local skills and resources. In general, policies could include incentivised payback periods from energy savings, such as those used in high-income countries, subsidises for materials and efficient appliances, improved housing guidelines and specialised support for homeowners, architects, designers, planners, and the DDA who are a major provider of new housing.

### Limitations and implications for future research

This paper represents an initial investigation into the energy use and health risks in housing for the case of Delhi, where there is little previous work or supporting data. Data limitations restrict the level of assessment detail and the accuracy of the results. While it was possible to aggregate the housing stock into four broad categories and describe general characteristics, it is not possible to breakdown the housing further into a set of archetypes and describe in detail their features, which would aid a more accurate estimate of energy use and health hazards. Similarly, energy use data, such as details of occupancy behaviour and appliance use, is restricted to only a couple of studies with limited scope. Additional data collection on housing characteristics in each settlement type is needed and surveys capturing appliance ownership, use and occupancy would provide a more accurate description of energy use across households. Our estimates at a stock level ignore any variation in appliance ownership; for example, we assume 100% penetration of air conditioning across the planned settlements, which is likely to be an oversimplification. It is recommended that national and state-wide surveys, such as the NSS, collect further data on household geometry, the composition of construction materials (beyond material type), and details on ventilation provision and detailed household energy use. This would enable the development of archetypes to establish a stock model that is representative of the housing in Delhi. Without this information, it is only possible to develop broad conclusions. Dukkipati et al. (2014) also recommend more appropriately designed surveys to increase data availability on energy use in India (Dukkipati *et al*. 2014).

Our modelled energy results were broadly in line with previous studies. However, these studies often take idealised or simulated cases, which may not reflect actual use. For example, Ramesh et al. (2012a, 2013) assume heating below 18°C and cooling above 25°C, which is not in line thresholds from thermal comfort studies (Ramesh *et al*. 2012a, 2013) and Mastrucci and Rao (2017) consider the energy required for ‘decent’ living to meet comfort needs (Mastrucci and Rao 2017). Studies with measured data do not clearly define appliance ownership or their usage, thus making it difficult to compare directly (Praseeda *et al*. 2016). Comparing the energy end-use in the planned dwelling with studies that include space cooling suggest similar trends with the highest energy use from cooling appliances, however, these studies do not consider other appliances such as TV and fridges (Mastrucci and Rao 2017) or do not provide adequate details of what other appliances were considered so direct comparisons are not possible (Ramesh *et al*. 2012a, 2013). More work is needed to assess actual energy use and interventions that help to protect for health across all housing groups to develop a better understanding of energy consumption and develop appropriate interventions. Furthermore, typically studies report metrics of energy use per unit floor area, which is useful highlighting efficiency in building performance but this is not appropriate for dwellings with vast differences in energy uses and floor area, and may provide misleading results.^1^ New metrics that demonstrate the disparities in energy use between house-holds, look beyond building performance and highlight energy use gaps in regards to health risks are vital to pinpoint where interventions should be targeted.

The methods used to assess the energy and health hazards across the settlement types are based on the best available data and expert opinion. Energy use estimates currently do not consider building performance, and variations in occupant behaviour and patterns, this would require in-depth data collection to develop these on a stock level. More sophisticated methods, such as building physics modelling, could provide better predictions of energy consumption as well as indoor environmental quality (hygrothermal conditions and exposure to pollutants). Health impact models could provide estimates of morbidity and mortality based on exposure-response functions. The level of data required for such methods is currently unavailable and significant further research is needed to gather this information. More work in this area is crucial to support effective policy to improve health and sustainability across housing stock in Delhi. Some consideration of the sensitivity and uncertainty in the applied methods is provided. The assessment of health hazards is carried out individually by the authors (variation of the response is provided in Appendix C) and then combined for the formulation of a final ranking, which helps to improve the objectivity of the analysis. A sensitivity analysis for energy use was performed for the planned archetype to assess the most influential parameter. This helped to identify where further detailed data is required as well as evaluate the variability of the overall results, however, further detailed surveying is required to provide the bounds of the parameters and to provide realistic estimates of the uncertainties.

Further data gathering work, the employment of more sophisticated methods and sensitivity analysis to assess variability in results will help to provide a more accurate assessment of health and energy in Delhi housing. However, this paper offers a starting point to focus on further research and helps identify missing data gaps. This assessment is vital in paving the way for more research and detailed investigation of the connections between housing, health and energy. Similar work could be carried out for other locations, as a first step, to raise the agenda of housing as potential means to improve health and sustainable and support progress towards the SDGs.

## Conclusions

We developed a framework for the assessment of housing energy use and health risks in low-income settings. The framework employed existing data sets and literature to assess the health risks and energy use across Delhi’s housing stock. The framework included the characterisation of the housing stock into archetypes, in this case, by settlement type, and then the assessment of these archetypes in regards to energy use and health risks, drawing on methods from the Built Environment and Public Health.

Despite limitations in data availability, our results show that energy use is nearly two times higher per occupant in planned dwellings compared to non-planned dwellings, as a result of higher ownership of A/C units. Health risks varied considerably across settlement types as a result of variations in the quality of housing, and the ability of occupants to modify their indoor environment. JJ clusters are most likely to be at a higher risk from a wide range of adverse health impacts compared to other settlement types. The greatest health risks, across all settlement types, were assessed to be from exposure to particulate matter, heat, and cold. We highlight the vital need for more data on this topic to enhance understanding of household energy use and health risks, which will help provide a more accurate understanding as well as support further research evaluating interventions.

This work forms a critical first step and can be used to develop guidelines for improving housing, helping to support pathways to an equitable, healthy, sustainable city. Further research should now be carried out to assess levels of exposure to the identified hazards and understand detailed energy use behaviour, as well as to assess intervention performance and trade-offs before implementation. The approach developed could be applied to other locations in India, South Asia and beyond to understand key priorities and interventions strategies that differ with varying housing and environmental risks.

## Supplementary Material

Appendices

## Figures and Tables

**Table 1:** Settlement types in Delhi, from (Government of National Capital Territory of Delhi 2008).

Type of Settlement	Development and Settlement Characteristics	Est. population in 2000 (million)	% of total est. population
JJ Clusters	Arose from encroachment on public/private land. Extremely poor living conditions.	2.072	14.8
Slum Designated Areas	Improved version of JJ Clusters	2.664	19.1
Unauthorised Colonies	Developed on agricultural land by illegal means	0.740	5.3
JJ Resettlement Colonies	Plots allocated by the DDA to resettle JJ clusters from 1975	1.776	12.7
Rural Villages	Will probably be urbanized by 2021. Similar characteristics to rural villages.	0.740	5.3
Regularised- Unauthorised Colonies	Similar characteristics to the unauthorized colony, but with better infrastructure and right to tenure.	1.776	12.7
Urban Villages	Rural villages that fell into urban areas after rapid urbanization	0.888	6.4
Planned Colonies	Planned by DDA or private agencies from the early 1960s.	3.308	23.7
Total:		13.964	100.00

**Table 2:** Typical properties of each settlement type.

Type:	Planned dwellings	Urban/rural villages	Unauthorised colonies	JJ Clusters	Ref
%	24	11	18	47	(Government of National Capital Territory of Delhi 2008)
Description:	- Planned housing built by private agencies or the DDA - Often high rise - Legal tenure & planned services	- Evolved organically over time, with legal tenure - Services introduced as and when without prior planning - 3 to 4 storey houses in close and narrow streets	- Built on illegal land however settlements are becoming regularised with legal tenure - Infrastructure is introduced on as and when basis - Little information on housing style	− 1 to 2 storey buildings, with small ground floor areas (20 m^2^ - Self-built and undergo incremental growth - Often only one façade exposed - No legal tenure, apart from in the case of JJ Resettlement colonies	(Sivam 2003, Ishtiyaq and Kumar 2011, UN Habitat, Kumar Soni 2011, Ahmad and Choi 2011, Mitchell 2010)
Rooms	1 x living room 2 x bedrooms 1 x kitchen 1 x bathroom	1 x living room 1 x bedroom 1 x kitchen 1 x bathroom	x living room x bedroom 1 x kitchen 1 x bathroom	2 x multi-purpose rooms	(Government of India 2011, Kumar Soni 2011, Mitchell 2010, Government of National Capital Territory of Delhi 2009)
Housing materials	- Wall: Plaster & Burnt Brick - Roof: Brick + Reinforced Cement Concrete	- As planned housing however indications suggest thicker roofs	- Little information but assumed to be as planned housing	Varied: from temporary building materials to brick and cement construction	(Government of India 2011, N. S. S. O.-M. of S. & P. 1. Government of India 2010a, Kumar and Suman 2013, Ramesh *et al.* 2010, TERI and Fraunhofer Institute for Building Physics)
Income distributions	- High/Mid-income groups	- Mid/Low-income groups	- Mid-income groups	- Low-income groups	(Government of National Capital Territory of Delhi 2008)
Cooking fuel and separate kitchen	- LPG - Separate kitchen	- LPG - Separate kitchen	- LPG - Separate kitchen	- LPG - No separate kitchen	(Government of India 2011)
Electrical appliances	- TV - Fridge - Lighting	- TV - Fridge - Lighting	- TV - Fridge - Lighting	- TV - Fridge - Lighting	(N. S. S. O.-M. of S. & P. 1. Government of India 2010a, TERI 2007, N. S. S. O.-M. of S. & P. 1. Government of India 2010b)
Ventilation and cooling systems Infrastructure and services	- AC & fans - Windows with cross ventilation likely	- Fans & air coolers - Poor levels of ventilation	- Fans & air coolers - Windows with cross ventilation likely	- Fans - Poor levels of ventilation (no or small windows)	(TERI 2007, N. S. S. O.-M. of S. & P. 1. Government of India 2010b)
Infrastructure and services	Piped water, toilets and sewage systems	Water tanks, toilets, containment tanks.	Water tanks, toilets, containment tanks	Water by tanker, no sanitation.	
Problems reported:	- High temperatures in top-floor flats	- Overcrowding, congestion, and structural dilapidation - Studies suggest reliance on artificial lighting and extremely poor levels of ventilation	No data available	- No available or low-quality infrastructure and facilities - Overcrowding, poor ventilation and tightly cramped housing	[Ishtiyaq and Kumar, 2011; Kumar Soni 2011; Mitchell, 2010; Nix E, et al., 2014]

**Table 3:** Assumptions on energy use in dwellings.

Category	Usage	Carbon Intensity
Cooking	13.3 kg LPG per month per household in all settlement types (D’Sa and Murthy 2004), assuming a calorific content of 45,750 kJ/kg (Natarajan *et al*. 2008)	0.2147 kg CO_2_ per kWh (Carbon Trust 2011)
Lighting	Estimated from (TERI 2007) to be: Bedrooms – 60 W bulbs 2hrs/day Living rooms 60 W bulbs 5hrs/day Bathrooms 55 W tube lighting 2hrs/day Kitchens 55 W tube lighting 2hrs/day	0.943 kG CO_2_ per kWh was assumed (IEA 2007)
Appliances	120 W TVs was calculated in all settlements 5hrs/day (TERI 2007) 200 W refrigerator was assumed to be always on (TERI 2007)	
Cooling	Fans (60 W) turned on in all dwellings when hourly external temperatures exceed 26.2°C during occupied hours in bedrooms and living rooms (Indraganti 2011). The external temperature was taken from a typical weather file for the location of Delhi, commonly used for building simulation (Indian Society of Heating Refrigerating and Air-Conditioning Engineers)	
	Air coolers (200 W) (used in unauthorised and urban villages) and A/C units (1750 W) (used in planned) turned on when external temperatures exceed 28.5 and 31.3°C respectively in occupied bedrooms and living rooms (Indraganti 2011)	

**Table 4:** Hazards assessed in the Delhi housing stock (from UK HHSRS apart from those marked * which were added for the Delhi stock).

Physiological requirements		
*Hygrothermal conditions, pollutants*	Psychological impacts *Space, security, light & noise*
Damp & mould	Overcrowding
Heat	Entry by intruders
Cold	Inadequate lighting
Particulate matter*	Noise
Asbestos	
CO and combustion products (NO_x,_ NO_2_ SO_2_)	
Uncombusted LPG		
Lead	
Radiation	
VOCs	
Infections	Accidents
*Hygiene, sanitation & water supply*	*Falls, electric shocks, fires, burns & scalds, collisions, cuts & strains*
Vector-borne diseases*	Falls baths
Domestic hygiene, pests, refuse	Falls level surfaces
Falls on stairs
Food safety	Falls between levels
Personal hygiene, sanitation and drainage	Electrical shocks
	Fire
Water supply	Flames, hot surfaces
	Collision, and entrapment
	Explosions
	Position and operability of amenities
	Structural collapse and falling elements

**Table 5:** Risk matrix used to assess each hazard based on the likelihood of occurrence and spread of harm.

	Likelihood of occurrence			
Expected harm	Low	Moderate	High	Severe
Low	1	2	3	4
Moderate	2	4	6	8
High	3	6	9	12
Severe	4	8	12	16

**Table 6:** Annual energy use by end-use (and percentages) by settlement type.

Energy use (kWh)			Settlement type			
Fuel type	End-use		Planned	Urban villages	Unauthorised	JJ clusters
LPG	Cooking		2028 (26%)	2028 (39%)	2028 (39%)	2028 (46%)
Electricity	Lighting		387 (5%)	234 (4%)	277 (5%)	153 (3%)
	Cooling appliances		3493 (44%)	1033 (20%)	963 (18%)	233 (5%)
	Other appliances		1971 (25%)	1971 (37%)	1971 (38%)	1971 (45%)
Total energy use			7879	5266	5239	4385

**Table 7:** Estimated household health hazard risks final rating (S_O,H_), with red denoting highest risk hazards and green lowest risk hazards (with modal responses of Low, Medium, High and Severe for the likelihood of occurrence, O, and expected harm, H, noted in subscript).

	Hazard	Settlement type			
		Planned	Urban villages	Unauthorised	JJ Clusters
Physiological requirements: Hygrothermal conditions, pollutants					
1	Damp & mould	4_M,M_	6_H,M_	4_M,M_	6H,M
2	Heat	6_M,H_	9_H,H_	6_M,H_	12_S,H_
3	Cold	6_M,H_	9_H,H_	6_M,H_	9_H,H_
4	Particulate matter	9_H,H_	9_H,H_	9_H,H_	16_S,S_
5	Asbestos	3_L,H_	3_L,H_	3_L,H_	6_M,H_
6	Biocides	2_L,M_	2_L,M_	2_L,M_	2_L,M_
7	CO and combustion products	_4M,M_	4_M,M_	4_M,M_	4_M,M_
8	Uncombusted LPG	_2L,M_	2_L,M_	2_L,M_	2_L,M_
9	Lead	2_L,M_	2_L,M_	4_M,M_	4_M,M_
10	Radon	3_L,H_	3_L,H_	3_L,H_	3_L,H_
11	VOCs	4_M,M_	1_L,L_	1_L,L_	1_L,L_
Psychological impacts: Space, security, light & noise					
12	Overcrowding	2_L,M_	6_H,M_	6_H,M_	6_H,M_
13	Entry by intruders	2_L,M_	2_L,M_	2_L,M_	2_L,M_
14	Inadequate lighting	1_L,L_	2_M,L_	2_M,L_	3_H,L_
15	Noise	2_L,M_	4_M,M_	4_M,M_	6_H,M_
Infections: Hygiene, sanitation & water supply					
16	Vector-borne disease	2_L,M_	6_M,H_	6_M,H_	9_H,H_
17	Domestic hygiene	2_L,M_	6_H,M_	4_M,M_	6_H,M_
18	Food safety	2_L,M_	4_M,M_	4_M,M_	6_H,M_
19	Personal hygiene, sanitation and drainage	2_L,M_	6_H,M_	4_M,M_	6_H,M_
20	Water supply	2_L,M_	6_H,M_	6_H,M_	9_H,H_
Accidents: Falls, electric shocks, fires, burns & scalds, collisions, cuts & strains					
21	Falls baths	1_L,L_	1_L,L_	1_L,L_	1_L,L_
22	Falls level surfaces	1_L,L_	2_M,L_	2_M,L_	2_M,L_
23	Falls on stairs	3_L,H_	4_M,M_	6_M,H_	6_M,H_
24	Falls between levels	2_L,M_	6_M,H_	3_L,H_	4_M,M_
25	Electrical shocks	2_L,M_	4_M,M_	4_M,M_	6_H,M_
26	Fire	3_L,H_	6_M,H_	3_L,H_	6_M,H_
27	Flames, hot surfaces	2_L,M_	4_M,M_	4_M,M_	6_H,M_
28	Collision, and entrapment	2_L,M_	2_L,M_	2_L,M_	2_L,M_
29	Explosions	4_L,S_	4_L,S_	4_L,S_	4_L,S_
30	Position and operability of amenities	1_L,L_	1_L,L_	1_L,L_	1_L,L_
31	Structural collapse and falling elements	3_L,H_	6_M,H_	3_L,H_	6_M,H_

## Notes

1 For example, if we assume average floor areas of 80, 40, 60 and 12 m^2^ for planned, urban village, unauthorised and JJ cluster dwellings respectively the annual energy use per unit area become 73, 81, 54, and 196kWh/m^2^. This suggests that the JJ clusters are least efficient, however, due to the very small floor area, this is a misleading result.
