# Supplementary material for: Housing, health and energy: a characterisation of risks and priorities across Delhi’s diverse settlements
Source: Cities Health. Author manuscript; Available in PMC 2024 Oct 15. (PMC7616699; doi:10.1080/23748834.2020.1800161)
Supplement: Appendices [file EMS199286-supplement-Appendices.pdf]

Appendices

Appendix A. Sensitivity analysis of energy use variables for the planned settlement archetype

A sensitivity analysis was conducted to understand the impact of input variables on energy use. This was carried out for the planned settlement archetype, which is most likely to use air conditioning. The input variables for power rating and hours of appliance use for each end-use were assumed to follow a normal distribution described by the mean and standard deviation. The mean for each variable was taken as the reported value in the literature, as described earlier in the paper. To account for the variation of each input, the standard deviation was calculated for each appliance from a range of ±20% the mean value. This was selected to account for a potentially wide variation in hours of use and appliance power ratings. Data is not currently available to describe the variation more accurately. The values used to describe each variable are presented in Table A1.

The probability distribution functions of each input variable were then used to generate a random sample to replicate the input variations expected. A sample size of 500 was used as this was deemed large enough so that sample mean does not change by more than 2%, as similar to carried out elsewhere (Das *et al.* 2014). This was done by generating a random number and then using the inverse cumulative distribution function to convert the generated random values into the domain of the input variables. The calculation for energy use was then completed for each permutation of the sample inputs.

To assess the effect of each parameter on the output, correlation-based and regression-based methods were used to measure the strength of the relationship between input and output variables. Pearson's Product Moment Correlation Coefficient was used to measure the strength of linear correlation between each input and output variable. R^2 coefficients were also calculated to assess the proportion of the variance that is predictable from the input variable. The coefficients for each variable are provided in Table A1.

Table A1. Sensitivity analysis input distribution and coefficients for energy use variables.

End-use		Input distribution		Sensitivity analysis method	
Type	Appliance	Mean	Standard deviation	Pearson Coefficient	R^2
Power rating (Watts)					
Lighting	Bulb	60	9.8	0.077	0.0059
	Tube	55	9.0	−0.017	0.00029
Other appliances	TV	120	19.6	0.018	0.00031
	Fridge	200	32.7	0.44	0.20
Cooling appliance	Fan	60	9.8	0.069	0.0047
	AC	1750	285.8	0.70	0.49
Hours of use (hours)					
Lighting	Bulb	5	0.8	0.013	0.00017
	Tube	2	0.3	−0.0094	0.000088
Other appliances	TV	5	0.8	0.077	0.0059
Cooling appliance (living room)	Fans	2881	470.5	0.12	0.015
	A/C	1450	236.8	0.58	0.33
Cooling appliance (bedroom)	Fans	999	163.1	0.020	0.00039
	A/C	140	22.9	0.10	0.010

Appendix B.

The following terms and descriptions were used by the authors to complete the health hazard risk analysis.
Definitions of terms:

- *Occurrence*: This is an event or period of time exposing an individual to a hazard.
- *Likelihood*: The probability of an occurrence that could cause harm and for this work it is to be assessed as the probability of an occurrence over a typical year.
- *Harm*: An adverse physical or mental effect on the health of a person, both permanent and temporary.
- *Expected harm*: The expected possible harm outcome, which could result from an occurrence.

Judging the likelihood of occurrence and expected harm:
For each given hazard the *Likelihood of occurrence* is assessed in regards to;

(i) the average likelihood of the hazard exposure (outdoor conditions, expected indoor conditions etc.)
(ii) the housing conditions/modifiers which may increase or reduce the likelihood of occurrence

and the *Expected harm* is assessed in regards to;

(i) the expected health effect (as described in the UKHHSRS) and health evidence for Delhi/India
(ii) the housing/settlement type conditions/modifiers or population demographics in given settlement type with may increase or mitigate the severity of the outcomes

Assessment categories:
Likelihood of occurrence is categorized as:

- *Low*: chance of occurrence is low and not expected to occur over an annual period
- *Moderate*: chance of occurrence is likely and expected to occur at least once during an annual period
- *High*: chance occurrence is highly likely and expected to occur more than once or over several days during an annual period
- *Severe*: chance occurrence is extremely likely and expected to occur for the majority of the annual period

Expected harm is categorized as:

- *Low*: no or limited harm to health expected (such as broken finger; slight concussion; moderate cuts to face or body; regular coughs or colds)
- *Moderate*: moderate harm to health expected (such as: hypertension; sleep disturbance; allergy; gastro-enteritis; diarrhoea; vomiting …)
- *High*: high harm to health expected (such as: asthma, respiratory diseases, lead poisoning, loss of hand or foot, serious burns …)
- *Severe*: severe harm to health expected (such as: death, lung cancer, permanent paralysis, permanent loss of consciousness; 80% burn injuries …)

Appendix C

Table C1 details the evidence used to generate the estimated likelihood of occurrence and spread of harm based on an assessment of exposure risk, housing modifiers/conditions and available health evidence. The ratings based on the metric developed in section 2.3.1 are also included. The mode (Mo), median (Md), maximum (Mx) and minimum (Mn) values from the individual responses are detailed here for each entry as a measure of the variability in *'expert opinion'*.

Table C1. Exposure risk, housing modifiers/conditions and available health evidence used to estimate the likelihood of harm and expected harm.

					Risk rating per settlement				
Hazard type	Health effects	Exposure risk	Housing modifiers/conditions	Health evidence	Type	Mo	Md	Mx	Mn
Physiological requirements: Hygrothermal conditions, pollutants									
Damp & mould	Asthma, allergic symptoms	- Outdoor RH can increase up to 80% in Delhi during the monsoon season.<br>- Measurements during the monsoon season in apartments in Hyderabad, which has a warm and humid climate, found an indoor RH of approximately 55% (Indraganti 2011), which is close to the 60% risk level for the UK above which of damp and mould growth becomes significant (Department For Communities and Local Government 2006).<br>- The research found high-exposure to fungi for Children in Delhi homes, with highest levels in winter months (Sharma *et al.* 2011).	- Lack of purpose-provided ventilation in both urban/rural villages and in JJ settlements may lead to higher indoor RH in these dwellings due to an inability to remove moisture produced from indoor activities.<br>- Rising damp through poorly constructed dwellings, particularly at risks are low-income dwellings built directly on the ground.	– 2.12% of recorded deaths in the NCT of Delhi from bronchitis and asthma (Government of National Capital Territory of Delhi 2012).<br>- High fungal counts were connected with a higher prevalence of skin sensitization and to respiratory allergies in Delhi Children (Sharma *et al.* 2011).	Planned<br>Urban villages<br>Unauthorised<br>JJ clusters	4<br>6<br>4<br>6	4<br>6<br>4<br>6	6<br>6<br>8<br>9	1<br>1<br>2<br>2
Heat	Cardiovascular stain (stroke), dehydration, respiratory conditions, genitourinary diseases	- Outdoor temperature up to 47°C in Delhi during the summer months.<br>- Indoor temperatures measured in the warm and humid climate regions of India range between 30–39°C during the summer months (Indraganti 2011, Singh *et al.* 2010, Hegde 2010, Dili *et al.* 2010).	- Limited penetration of air conditioning and limited purpose provided natural ventilation, high occupant density, and a lack of climate-sensitive design features in the self-built JJ cluster structures (Mitchell 2010, TERI 2007).<br>- Modifications such as partitioning of structures into multi-unit dwellings in urban/rural villages could lead to overheating in the summer due to a combination of overcrowding and reduced cross-ventilation potential (Kumar Soni 2011).<br>- Planned dwellings are likely to have access to air conditioning with top floor flats shown to be most reliant on A/C use in an observational study carried out in apartments in Hyderabad (Indraganti 2011).	- Heatwave mortality has been increasing (Akhtar 2007) with the highest mortality burden in the poorest states (Kumar 1998).<br>- In Delhi, 3.9% increase in mortality for each degree increase above mean daily temperature 29°C (lags of 0–1 days) (McMichael *et al.* 2008).<br>- EM-DAT database lists 25 heatwaves recorded between 1953 to 2015, resulting in 11,926 deaths across India (Guha-Sapir *et al.*)	Planned<br>Urban villages<br>Unauthorised<br>JJ clusters	6<br>6<br>6<br>12	6<br>6<br>9<br>12	6<br>9<br>12<br>12	2<br>6<br>6<br>9
Cold	Cardio-respiratory illness (including heart attack, stroke, upper and lower respiratory tract infection)	- Minimum outdoor temperatures in winter have been recorded to be 0°C.<br>- Indoor temperatures measured in the warm and humid climate regions of India fell below 19°C during the winter months (Indraganti 2011, Singh *et al.* 2010, Hegde 2010, Dili *et al.* 2010).	- Little evidence of the use of heating systems.<br>- Leaky buildings are unable to control heat loss.	– 2.12% of recorded deaths in the NCT of Delhi from bronchitis and asthma, 1.54% from pneumonia, 0.17% from influenza (Government of National Capital Territory of Delhi 2012).<br>- In Delhi, 3.9% increase in mortality for each degree increase below mean daily temperature 19°C (lags of 0–1 days) (McMichael *et al.* 2008).<br>- EM-DAT database lists 29 cold waves recorded between 1961 to 2015, resulting in 5268 deaths across India and 2 occurrences of severe winter conditions with 320 deaths (Guha-Sapir *et al.*).	Planned<br>Urban villages<br>Unauthorised<br>JJ clusters	6<br>9<br>6<br>9	6<br>6<br>6<br>9	6<br>9<br>9<br>9	1<br>2<br>4<br>4

(*Continued*)

Table C1. (Continued).

Hazard type	Health effects	Exposure risk	Housing modifiers/conditions	Health evidence	Risk rating per settlement				
					Type	Mo	Md	Mx	Mn
Particulate matter (indoor and outdoor)	Cardiopulmonary disease, lung cancer, asthma. other	- Monthly mean indoor concentrations of PM (from indoor and outdoor sources combined) were found to vary between 56–106 μg/m^3 and 152–201 μg/m3 for PM2.5 and PM10 respectively in two Delhi dwellings (Khillare *et al*. 2004).<br>- A study in 14 residential dwellings in neighbouring Agra found a six-month (between October and March) mean of 135–173 μg/m3 for PM2.5 (Massey *et al*. 2009). These concentrations are far higher than values in outdoor air quality guidance provided by the WHO (WHO 2010) for PM2.5 (maximum annual mean of 10 μg/m3 and 24-hour mean of 25 μg/m3) and PM10 (maximum annual mean of 20 μg/m3 and 24-hour mean of 50 μg/m3).<br>among men and low-income groups (Rani *et al*. 2003).<br>- Indoor sources from cooking. Lighting and heating can also contribute to PM levels.	- Poor-quality structures with very permeable envelopes in urban/rural villages and in JJ settlements put occupants most at risk.<br>- Tighter dwellings and those utilising air-conditioning systems where windows remain closed, such as in the planned settlements, will inhibit the ingress of outdoor pollutants.<br>- 20% of occupants, mainly in JJ clusters, do not have separate kitchens (Government of India 2011), and most dwellings do not have ventilation systems for effective removal of pollutants during cooking times (e.g. through a chimney or an extract fan).<br>- Smoking indoors significantly influences indoor PM levels (Slezakova *et al*. 2009), and smoking rates in India are high (30% of those aged 15 or higher), especially among men and low-income groups (Rani *et al*. 2003).	- Smith estimated the total annual number of premature deaths from indoor air pollution among children below the age of five and adult women is between 400,000 and 550,000 in India (Smith 2000).<br>- 2.12% of recorded deaths from bronchitis and asthma, 6.16% from cancer, 11.21% from heart diseases and heart attacks in the NCT of Delhi (Government of National Capital Territory of Delhi 2012).<br>- SPM levels found to be significant to asthma prevalence amongst children in Delhi households (Kumar *et al*. 2015).<br>- Self-reported health problems as result from indoor air pollution included acute respiratory infections (p-value < 0.001), throat, eye and skin infections (p-value = 0.02), asthma (0.005)	Planned	12	6	12	2
					Urban villages	12	9	12	4
					Unauthorised	9	12	16	9
					JJ clusters	16	16	16	9
Asbestos	Pleural and lung cancer, mesothelioma, asbestosis	- Up to 11% of dwellings in Delhi (Government of India 2011) use asbestos sheets as the predominant material of the roof. If these materials are damaged in any way, they release fibres that are dangerous for health.	- The use of asbestos sheets is most likely in the low-income JJ clusters settlements, where roofing materials are more diverse (Government of National Capital Territory of Delhi 2009).	6.16% recorded deaths from cancer in the NCT of Delhi (Government of National Capital Territory of Delhi 2012).	Planned	3	3	4	1
					Urban villages	1	3	6	1
					Unauthorised	3	3	12	1
					JJ clusters	6	6	16	1
Biocides	Dependent on biocide	*Insufficient data*	*Insufficient data*	*Insufficient data*	Planned	2	2	2	1
					Urban villages	2	2	2	1
					Unauthorised	2	2	4	1
					JJ clusters	2	2	4	1
CO and combustion products (indoor and outdoor) (NOx, NO2 SO2)	Headaches, nausea, damage of airway linings, bronchitis, death	- Indoor concentrations of CO, SO_2, NO_x, and NO_2 measured in neighbouring Agra were found to be below maximum values given in standards produced by India's Central Pollution Control Board (Lawrence *et al*. 2005).<br>- Almost all dwellings in Delhi use LPG, oil, and solid fuels containing carbon for cooking and 0.7% of dwellings use kerosene, other oil, or 'any other' type of fuel for lighting (Government of India 2011). These fuels are sources of PM, NO2, SO2, and CO in the case of incomplete combustion.	– 20% of occupants, mainly in JJ clusters, do not have separate kitchens (Government of India 2011), and most dwellings do not have ventilation systems for effective removal of pollutants during cooking times (e.g. through a chimney or an extract fan).	2.12% of recorded deaths from bronchitis and asthma in the NCT of Delhi (Government of National Capital Territory of Delhi 2012)<br>- NO2 and SO2 found insignificant to asthma prevalence amongst children in Delhi households (Kumar *et al*. 2015).	Planned	4	3	4	1
					Urban villages	4	4	6	1
					Unauthorised	4	4	9	2
					JJ clusters	4	6	16	2
Uncombusted LPG	Asphyxiation	- The use of LPG also presents a risk as it may escape uncombusted into a dwelling due to defects in the installation or appliance.	- Uncombusted LPG could be	*Insufficient data*	Planned	2	3	4	1
					Urban villages	4	3	4	1
					Unauthorised	2	2	9	1
					JJ clusters	2	3	9	1

(*Continued*)

Table C1. (Continued).

Hazard type	Health effects	Exposure risk	Housing modifiers/conditions	Health evidence	Risk rating per settlement				
					Type	Mo	Md	Mx	Mn
Lead	Neural development and other effects	- Pb elements found in the characterization of ambient $PM_{2.5}$ (Khillare *et al.* 2004, Pant *et al.* 2015), with maximum concentrations up to 2.51 $\mu g/m^3$ in a pollution hotspot (Pant *et al.* 2015)<br>- Indoor/outdoor ratios of Pb concentration found to be 0.91–0.97 for two sites in Delhi (Khillare *et al.* 2004), with higher indoor concentrations correlated to road proximity (Kumar 2001).	*Refer to housing modifiers for particulate matter*	*Insufficient data*	Planned	2	2	4	1
					Urban villages	4	2	4	1
					Unauthorised	2	2	6	2
					JJ clusters	4	4	6	2
Radon	Lung cancer	- One study found indoor radon levels to be below an action level of 200 Bq/m3 (based on recommendations in from the International Commission on Radiological Protection[2]) in Delhi (College 2012),	- Ventilation will further impact radon levels, more research is needed to fully assess this issue across a range of dwelling types.	6.16% recorded deaths from cancer in the NCT of Delhi (Government of National Capital Territory of Delhi 2012)	Planned	3	3	4	1
					Urban villages	3	3	4	1
					Unauthorised	3	3	6	2
					JJ clusters	3	3	6	2
VOCs	Allergic reactions, headaches, nausea, drowsiness	- High concentrations of volatile organic compounds (VOCs) in ambient air found in Delhi (Srivastava and Majumdar 2010).	- Volatile Organic Compounds (VOCs) may be found in a variety of materials in the home, with newly built dwellings most likely to be most affected, due to the higher emission rates of VOCs in any new materials such as carpet and paint.<br>- For exposure outdoor refer to housing modifiers for particulate matter	*Insufficient data*	Planned	2	2	4	2
					Urban villages	2	2	2	1
					Unauthorised	2	2	2	1
					JJ clusters	2	2	2	1
Psychological impacts: Space, security, light & noise									
Overcrowding	Increased heart rate and irritability. Increased hygiene risks, accidents, and contagious disease	- Delhi dwellings are at severe risk of overcrowding with a most common occupancy of 6–8 people (26%, (Government of India 2011)) combined with most commonly only one room (32%, (Government of India 2011)).	- Overcrowding has been reported in both the urban/rural villages and JJ cluster settlements (Ishtiyaq and Kumar 2011, Mitchell 2010).	- Overcrowding self-reported to have significant effect on: other common diseases (including headache, nausea, fever and vomiting) (p-value = 0.05); acute respiratory conditions (p-value <0.001); asthma (p-value = 0.03) and tuberculosis (p-value <0.001).	Planned	2	2	4	1
					Urban villages	6	6	9	2
					Unauthorised	6	6	9	4
					JJ clusters	6	8	9	6
Entry by intruders	Stress, injuries	- In 2012, there were 1715 recorded burglaries, up by 20% on the previous year; however, this number appears to fairly low compared to the number of households in Delhi. We assume it likely that many crimes go unrecorded, and there is a high risk of burglary in Delhi households, which could lead to anxiety or injury, in the case of aggregated burglary, to occupants	*Insufficient data*	*Insufficient data*	Planned	2	2	2	2
					Urban villages	2	2	6	1
					Unauthorised	2	2	4	1
					JJ clusters	4	4	6	1
Inadequate lighting	Depression, eye strain	- Inadequate lighting is likely to be an issue, especially during hot periods where curtains are kept drawn to keep the heat out. At times of power cuts and low voltage, lighting levels may fluctuate.	- Studies in an urban village noted inadequate daylight levels and reliance on artificial lighting (Kumar Soni 2011).	*Insufficient data*	Planned	1	1	2	1
					Urban villages	2	2	6	1
					Unauthorised	2	2	6	2
					JJ clusters	3	3	6	2

(*Continued*)

Table C1. (Continued).

Hazard type	Health effects	Exposure risk	Housing modifiers/conditions	Health evidence	Risk rating per settlement: Type	Mo	Md	Mx	Mn
Noise	Irritability, sleep disturbance, headache	- Noise is also likely to be an issue due to overcrowding, high population, dense housing infrastructure, and a high volume of traffic.	*Insufficient data*	A study relating noise pollution to self-reported human health conditions in Delhi (Firdaus and Ahmad 2010) in both a high-density area and a low-density area found a range of adverse health effects including nausea, rise in blood pressure, and depression as a result of factors including vehicles, generators, and household industries.	Planned	2	2	4	1
					Urban villages	4	4	6	2
					Unauthorised	4	4	6	1
					JJ clusters	6	4	6	3
Infections: Hygiene, sanitation & water supply									
Vector-borne disease	Malaria, Dengue fever, Japan	- Vector-borne disease is infections transmitted by the bite of infected arthropod species, such as mosquitoes, ticks, triatomine bugs, sandflies, and blackflies. In India, these risks include: Malaria, Dengue, Lymphatic Filariasis, Kala-azar, Japanese Encephalitis and Chikungunya	- Uncontrollable ventilation, over-occupancy, and inadequate sanitation systems in JJ clusters and urban/rural village dwellings make them particularly vulnerable to the spread of vector-borne diseases (VBDs).<br>– Transmission by direct contact with an infected individual or contaminated surface could be exacerbated in overcrowded dwellings.<br>-Airborne pathogens would be strongly influenced by overcrowding and ventilation rate, with a possible dependence on indoor air conditions like RH and temperature (Li *et al.* 2007).	- The major VBDs in India and their estimated health burdens in 2008 were; malaria (1,524,939 cases with 935 deaths), dengue (12,561 cases with 80 deaths), chikungunya (2,461–95,091 cases), filariasis (26,702 cases), Japanese encephalitis (3,839 cases with 684 deaths) and visceral leishmaniasis (33,234 with 146 deaths) (Dhiman *et al.* 2010), although, apart from dengue, these are more common in rural than urban areas.<br>- 0.30% deaths recorded due to malaria (Government of National Capital Territory of Delhi 2012).	Planned	4	3	6	2
					Urban villages	6	6	9	4
					Unauthorised	4	6	9	4
					JJ clusters	9	9	12	6
Domestic hygiene, pests, refuse	Gastrointestinal disease, asthma, allergic reactions	- Waste collection in Delhi is inadequate; it is reported that around 30% municipal solid waste (MSW) is left uncollected on the street or in small open dumps, and where collected MSW is dumped in uncontrollable open landfill causing a risk to both environment and human health (Talyan *et al.* 2008). Waste can cause pest infestations, which can then cause allergic reactions or carry infectious diseases, further heightening health risks.	- Planned and unauthorised housing tend to have better-managed surroundings or are situated in gated grounds, lowering the contamination potential of some solid waste and some pests, such as feral dogs.<br>- Urban/rural villages and JJ clusters often share street access and surrounding areas can be littered with waste and home to many pests.	– 0.02% of recorded deaths in NCT of Delhi from Rabies, 0.03% Diphtheria, 2.77% tetanus (Government of National Capital Territory of Delhi 2012).	Planned	4	2	4	1
					Urban villages	6	6	12	2
					Unauthorised	4	4	4	2
					JJ clusters	6	6	12	4
Food safety	Food poisoning, gastro-intestinal disease,	- Could be caused by contaminated food or inadequate storage of food. 56% of households are reported to have ownership of a refrigerator (N. S. S. O.-M. of S. & P. I. Government of India 2010b).	- JJ clusters are likely to be most at risk of food contamination due to the overcrowded dwelling with inappropriate storage space or poor hygiene from multi-use surfaces. Ownership of refrigerators lowest among low-income groups (N. S. S. O.-M. of S. & P. I. Government of India 2010b).	– 0.01% recorded deaths from food poisoning, 0.21% dysentery & diarrhoea. 0.03% diphtheria (Government of National Capital Territory of Delhi 2012).	Planned	2	2	4	2
					Urban villages	4	4	6	2
					Unauthorised	4	4	4	2
					JJ clusters	6	6	9	4

(*Continued*)

Table C1. (Continued).

Hazard type	Health effects	Exposure risk	Housing modifiers/conditions	Health evidence	Risk rating per settlement: Type	Mo	Md	Mx	Mn
Personal hygiene, sanitation and drainage	Gastrointestinal disease, skin infections, dysentery	- Sanitation infrastructure in Delhi is limited – 40% of dwellings are reported to have open drains and 7% have no drainage arrangement at all (Government of National Capital Territory of Delhi 2009).<br>- 21% of households are recorded to have no bathroom, with the majority of these households in low-income groups, 63% of households have exclusive use of a latrine, 22% have access to a shared latrine, and 9% have access to public latrines (Government of National Capital Territory of Delhi 2009), further suggesting facilities are limited.	- JJ clusters have the poorest sanitation infrastructure, often with no latrine facilities and substandard open drainage systems. Similarly, urban village infrastructure is haphazard, and as such, drainage and sanitation facilities can be poor (Ishtiyaq and Kumar 2010).	– 0.21% deaths from dysentery & diarrhoea, 0.48% from cholera. 0.03% typhoid, tetanus 2.77%, tuberculosis 3.34%, 0.03% diphtheria (Government of National Capital Territory of Delhi 2012).	Planned	2	2	4	1
					Urban villages	6	4	6	2
					Unauthorised	4	4	9	1
					JJ clusters	9	6	9	4
Water supply	Dehydration, gastro-intestinal disease, legionella	- According to survey data, the majority (84%) of households have access to tap water as a first drinking source, however, only 60% have exclusive use, with nearly 40% relying on shared or community sources (Government of National Capital Territory of Delhi 2009).<br>- Delhi's water supply can be contaminated, with outbreaks of waterborne diseases common (Ministry of Environment and Forests Power Government of India 2001), leading to further risk of illness.	- The low-income groups are most likely to suffer from an inadequate water supply, and JJ clusters are likely to be at the highest risk (Government of National Capital Territory of Delhi 2009).	0.21% dysentery & diarrhoea of all recorded deaths (Government of National Capital Territory of Delhi 2012).	Planned	4	2	4	1
					Urban villages	6	6	9	2
					Unauthorised	6	6	9	1
					JJ clusters	9	9	9	4
Accidents: Falls, electric shocks, fires, burns & scalds, collisions, cuts & strains									
Falls baths	Lacerations, fractures, heart attack, death	- Poor design of bath or mobility of the occupant with heightened hazard.	- Little information about falls in baths but assumed to be the same in all settlement types where baths are available.	0.81% falls and drowning of all recorded deaths (Government of National Capital Territory of Delhi 2012).	Planned				
					Urban villages	1	1	2	1
					Unauthorised	1	2	6	1
					JJ clusters	1	2	6	1
Falls level surfaces	Lacerations, fractures, heart attack, death	- Level surfaces badly finished with large open drains causing heightened hazards.	- Likely to be the case in informally constructed rural/urban villages and JJ clusters.	0.81% falls and drowning of all recorded deaths (Government of National Capital Territory of Delhi 2012).	Planned	1	2	6	1
					Urban villages	2	2	6	1
					Unauthorised	4	3	9	1
					JJ clusters	2	2	9	2
Falls on stairs	Lacerations, fractures, heart attack, death	- Poorly designed stairs and stairs without hand-rails and barriers likely to heighten hazards.	- Likely to be greater in JJ clusters and urban/villages where stairs are often narrow and built on the outside of dwellings, heightening risks if falls occur.	0.81% falls and drowning of all recorded deaths (Government of National Capital Territory of Delhi 2012).	Planned	2	3	9	2
					Urban villages	3	3	4	1
					Unauthorised	4	4	8	1
					JJ clusters	2	3	6	2
Falls between levels	Lacerations, fractures, heart attack, death	- Poorly designed housing or inadequate provision/quality of barriers/rails.	- Dwellings are self-built and often open-fronted denoting a possibility of risk of falling between levels.	0.81% falls and drowning of all recorded deaths (Government of National Capital Territory of Delhi 2012).	Planned	6	6	12	2
					Urban villages	2	3	6	1
					Unauthorised	8	4	8	2
					JJ clusters	4	4	9	1

(*Continued*)

Table C1. (Continued).

Hazard type	Health effects	Exposure risk	Housing modifiers/conditions	Health evidence	Risk rating per settlement: Type	Mo	Md	Mx	Mn
Electrical shocks	Disruption of normal heartbeat/ respiratory muscles, burns, death	- The risk from electrical shocks could arise from unsuitable and badly-installed electrical fittings.	- More likely in low-income households such as JJ clusters, where domestic wiring is temporary or fixed to walls rather than in conduit casing with adequate protection (Government of National Capital Territory of Delhi 2009).	1.36% deaths from accidental burns (Government of National Capital Territory of Delhi 2012).	Planned	6	6	12	2
					Urban villages	3	3	4	2
					Unauthorised	6	6	6	2
					JJ clusters	4	4	12	2
Fire	Burns, death	- Risk of fire could also result from the burning of fuels for cooking, lighting or heating in all settlements.	- In tightly packed JJ clusters or urban/rural villages, the risk of the fire spreading is higher.-	– 1.36% deaths from accidental burns (Government of National Capital Territory of Delhi 2012).<br>- EM-DAT database lists 29 fire occurrences between 1978 to 2015, resulting in 1655 deaths and 50,090 people affected across India (Guha-Sapir *et al.*).	Planned	6	6	12	2
					Urban villages	3	3	4	1
					Unauthorised	6	6	12	2
					JJ clusters	3	4	12	3
Flames, hot surfaces	Burns, death	- The risk for burns from flames and hot surfaces due to cooking, heating or lighting.	- Risks could be higher in cramped dwellings, where the occurrence of accidents are likely to be higher.<br>- 64.9% of all burn admissions were from families living in a single room dwelling unit and 34.3% of admissions from families having two rooms in the dwelling unit and floor level cooking resulted in the majority of accidents.	– 1.36% deaths from accidental burns. Burn deaths could also be a result of septicaemia 4.79% (Government of National Capital Territory of Delhi 2012).<br>- In the Lok Nayak Hospital during 01/01/09 – 31/05/10, 731 out of 991 burn ward patients (73.7%) were flame burns (kerosene, LPG, petrol, coal etc.), 95% in the home.<br>- 56.5% of LPG burn patients were discharged, 33.3% patients expired. 45.3% of kerosene burns discharged, 50.6% expired.	Planned	6	6	12	4
					Urban villages	2	2	4	2
					Unauthorised	4	4	6	2
					JJ clusters	4	4	6	2
Collision, and entrapment	Injuries, fractures, death	- Poor design of openings such as windows/doors and other features.	*Insufficient data*	0.02% deaths from other accidents (Government of National Capital Territory of Delhi 2012).	Planned	6	6	9	4
					Urban villages	2	2	3	1
					Unauthorised	2	2	4	1
					JJ clusters	2	2	3	1
Explosions	Injuries, fractures, death	- Use of canisters for LPG use could risk explosions.	*Insufficient data*	- EM-DAT database lists 10 non-industrial explosions between 1990 to 2015, resulting in 301 deaths across India (Guha-Sapir *et al.*).	Planned	2	2	4	1
					Urban villages	4	4	4	3
					Unauthorised	4	4	6	3
					JJ clusters	4	4	6	3
Position and operability of amenities	Strain and sprain injuries	- Poor design leading to physical strain associated with functional space and other features at dwellings.	*Insufficient data*	*Insufficient data*	Planned	4	4	6	3
					Urban villages	1	1	2	1
					Unauthorised	1	1	2	1
					JJ clusters	1	1	2	1
Structural collapse and falling elements	Injuries, fractures, death	- Poor structural quality causing whole-dwelling collapse, or of an element or a part of the fabric being displaced or falling.<br>- The area is at risk from earthquakes as such dwelling should have an appropriate structure.	- The risk from structural collapse and dilapidation is likely to be higher in both urban/rural villages where there is a lack of maintenance and JJ settlements where structures are often self-built without formal standards (Ishtiyaq and Kumar 2011, Mitchell 2010).	- EM-DAT database lists 43 non-industrial collapse occurrences between 1967 to 2015, resulting in 2941 deaths and 150,000 people affected across India (Guha-Sapir *et al.*).	Planned	1	1	2	1
					Urban villages	3	3	4	1
					Unauthorised	6	6	12	4
					JJ clusters	6	6	12	3
